# Before and after implementation of group antenatal care in Rwanda: a qualitative study of women’s experiences

**DOI:** 10.1186/s12978-019-0750-5

**Published:** 2019-06-27

**Authors:** Angele Musabyimana, Tiffany Lundeen, Elizabeth Butrick, Felix Sayinzoga, Bernard Ngabo Rwabufigiri, Dilys Walker, Sabine F. Musange

**Affiliations:** 1 School of Public Health, College of Medicine and Health Sciences, University of Rwanda, P.O Box 3286, Kigali, Rwanda; 2 Institute for Global Health Sciences, University of California, San Francisco, 550 16th Street, 3rd floor, San Francisco, CA 94158 USA; 3 Maternal, Child and Community Health Division, Rwanda Biomedical Center, Kigali, Rwanda

**Keywords:** Rwanda, Antenatal care, Prenatal care, Pregnancy, Postnatal care, Group care, Qualitative study, Clients’ perspectives, Sub-Saharan Africa

## Abstract

**Background:**

The Preterm Birth Initiative-Rwanda is conducting a 36-cluster randomized controlled trial of group antenatal and postnatal care. In the context of this trial, we collected qualitative data before and after implementation. The purpose was two-fold. First, to inform the design of the group care program before implementation and second, to document women’s experiences of group care at the mid-point of the trial to make ongoing programmatic adjustments and improvements.

**Methods:**

We completed 8 focus group discussions among women of reproductive age before group care implementation and 6 focus group discussions among women who participated in group antenatal care and/or postnatal care at 18 health centers that introduced the model, approximately 9 months after implementation.

**Results:**

Before implementation, focus group participants reported both enthusiasm for the potential for support and insight from a group of peers and concern about the risk of sharing private information with peers who may judge, mock, or gossip. After implementation, group care participants reported benefits including increased knowledge, peer support, and more satisfying relationships with providers. When asked about barriers to group care participation, none of them cited concern about privacy but instead cited lack of financial resources, lack of cooperation from a male partner, and long distances to the health center. Finally, women stated that the group care experience would be improved if all participants and providers arrived on time and remained focused on the group care visit throughout.

**Discussion:**

These results are consistent with other published reports of women’s perceptions of group antenatal care, especially increased pregnancy- and parenting-related knowledge, peer support, and improved relationships with health care providers. Some results were unexpected, especially the consequences of staff allocation patterns that resulted in providers arriving late for group visits or having to leave during group visits to attend to other facility services, which diminished women’s experiences of care.

**Conclusion:**

Group antenatal and postnatal care provide compelling benefits to women and families. If the model requires the addition of human resources at the health center, intensive reminder communications, and large-scale community outreach to benefit the largest number of pregnant and postnatal mothers, those additional resources required must be factored into any future decision to scale a group care model.

**Trial registration:**

This trial is registered at clinicaltrials.gov as NCT03154177.

**Electronic supplementary material:**

The online version of this article (10.1186/s12978-019-0750-5) contains supplementary material, which is available to authorized users.

## Plain English summary

Group antenatal and postnatal care is a different way of organizing health care visits during and after pregnancy. In group care, pregnant women are organized into groups and they attend all their visits at the health center together while participating actively in their health assessments and learning discussions. We studied Rwandan women’s opinions about group care during pregnancy before and after starting a group antenatal care program in 18 Rwandan health centers. We convened 8 focus groups with 84 women who had recently given birth before the group care program started to ask them what they thought of the group care concept. About 9 months after the program started, we convened 6 focus groups with 56 women from health centers randomized to group care.

We learned that Rwandan women were enthusiastic about the idea of group care because they perceived it would increase information-sharing and peer support during pregnancy, but that they also worried about the potential for their personal information to be shared outside the group and the negative consequences associated with reductions in their personal privacy loss. Women who participated in group care said they had learned much more about pregnancy, birth and motherhood than they expected and enjoyed closer relationships with other mothers and their care providers. However, women also reported difficulties that decreased their participation in group pregnancy care, such as lack of financial resources, lack of cooperation from the male partner, and long travel distances from home to health center. They also suggested that providers should not be interrupted during scheduled group care visits. These results will help stakeholders plan for future group care programs in Rwanda.

## Background

### Utilization of antenatal care and postnatal care in Rwanda

The 2015 Rwanda Demographic and Health Survey reported that 99% of women attended at least one ANC visit, while 52% attended either 2 or 3 visits and 44% attended at least 4 ANC visits [1]. In the Rwanda health system, Community Health Workers encourage women to enter ANC, which is provided at health centers; once registered at the health center, women with any risk factors are referred to the district hospital for evaluation. There is at least one health center in each administrative sector that provides a package of basic services, including routine antenatal care and uncomplicated vaginal birth and postnatal care [2]. In 2003, Rwanda implemented the focused antenatal care (FANC) strategy advocated by the World Health Organization before 2016 [3, 4]; women are encouraged to attend 4 total ANC visits but the timing of each visit must be according to the FANC outline. ANC services are most commonly provided by a nurse [1]. Women are not reminded to attend ANC visits by phone but are given a written appointment date for their next visit; a Community Health Worker at the village level ideally reminds pregnant women to attend ANC and postnatal care (PNC). Eighty-four percent of Rwanda’s residents live in rural areas and 69% of women report farming as their occupation [1, 5].

Other publications have reported on factors that are associated with low ANC utilization. A secondary analysis of the 2010 Rwanda Demographic and Health Survey reported that long distance between home and health center, having 4 or more children, and unwanted/unplanned pregnancy were associated with decreased ANC utilization [6]. Other reported barriers to ANC utilization in Rwanda include cultural norms, such as avoiding a public disclosure of pregnancy until the second trimester (when the pregnancy can no longer be easily hidden by clothing), and negative experiences with ANC providers, such as being criticized for registering for ANC too early or too late or presenting without a male partner [7].

Antenatal care in Rwanda is not provided to pregnant women for free. The health care system is partly financed with a universal, community-based health insurance program (“Mutuelle de santé”) established by the Government of Rwanda and formalized by law in 2008 [8]. This program uses a policy of annual household subscription, tiered payment levels by income, and co-payments (for all except the lowest-income-level clients) to the health center at the time of service, including for routine ANC. The co-payment required for one follow-up ANC visit is commonly 200 Rwandan Francs (0.25 USD, 2019) and the total estimated household cost for all ANC services was recently reported as approximately 3000 Rwandan Francs [5]. It is not known what effect these costs have on ANC utilization, but some published qualitative data suggest that confusion, apprehension, or misperceptions about health insurance coverage may dis-incentivize some women [7].

A comprehensive postnatal care (PNC) program continues to develop in Rwanda. The 2014–2015 Demographic and Health Survey reported that while 91% of women delivered in a health facility, only 19% of newborns had a postnatal checkup within 2 days of birth and 43% of women reported a postnatal checkup within 2 days of birth [1]. In 2015, the Rwanda Ministry of Health defined a four-visit postnatal care package meant to monitor the well-being of all newborns and mothers [9]. The first postnatal visit is to be accomplished by a provider (nurse or midwife), usually assigned to the maternity service at the health center, before discharge home from the facility after birth. The second and third postnatal visits are to be accomplished by a Community Health Worker in the home at 2 days and between 3 and 7 days following delivery. The fourth postnatal visit should be accomplished at the health center by a skilled provider at approximately 6 weeks after birth. National implementation of this postnatal care package is ongoing.

In 2015, the East Africa Preterm Birth Initiative-Rwanda, a partnership among the Rwanda Ministry of Health, the Rwanda Biomedical Center, the University of Rwanda School of Public Health, and the University of California, San Francisco, Institute of Global Health Sciences, decided to implement a cluster randomized controlled trial of group ANC and group PNC powered to assess the impact of this model on gestational age at birth in 5 districts. The design and objectives of this study are described elsewhere [10]. In group ANC, women are organized into groups and attend their ANC visits together over the course of pregnancy. Group visits are facilitated by skilled providers and include both health assessments and discussion with active participation by all group members. Several review articles offer summaries of outcomes associated with group ANC, which have included, in select populations, decreased rates of preterm birth and increased levels of satisfaction with care [11–14]. The potential to realize improved perinatal outcomes in Rwanda through this innovative service delivery model inspired these partners to implement a group ANC /PNC package as the standard of care at 18 pair-matched and randomized health centers in 5 districts during an 18-month trial period.

In 2016, the World Health Organization published an updated set of ANC recommendations that included several references to group ANC as an alternative model of care that deserves rigorous research [15]. Published quantitative data suggest that group ANC is enthusiastically received in the LMIC contexts in which it is introduced, although the rate at which women decline to participate is not known [16–21]. In the context of planning and conducting a 36-cluster randomized controlled trial of group ANC and group PNC in Rwanda, qualitative data was gathered among women before and after implementation. The purpose of this data collection was two-fold: first, to inform the design of the group care program before implementation; and second, to document women’s experiences of group care at the mid-point of the trial to make ongoing programmatic adjustments and improvements. We hoped to understand what women’s perceptions of the group ANC/PNC model were before implementation so we could align the program with their desires, both in terms of services offered and communication in the community about those services. After implementation, we wanted to understand whether there were any particular barriers to participation in group ANC/PNC that could be addressed at the facility, community, and policy levels.

## Methods

### Study design and setting

This qualitative study was done in conjunction with the East Africa Preterm Birth Initiative trial of Group Antenatal and Postnatal Care in Rwanda. Two rounds of qualitative data collection were completed. Round one, conducted in August 2016, included 16 focus group discussions (FGDs) and 22 in-depth interviews with providers and health officials. Round 2 consisted 6 FGDs and was completed in early April 2018, approximately 9 months after the group model was implemented. This paper will report only on FGDs with women about their perceptions of group antenatal and postnatal care before and after group care implementation.

FGDs for both rounds were conducted in Kinyarwanda, the local language, and audio-recorded. Participants were seated in a semi-circle to facilitate discussion and each participant was labeled with a number to allow the note-taker to identify who was speaking while maintaining confidentiality. FGDs ranged from 90 to 120 min in duration. At the end of each day, a debriefing meeting with the field team was conducted to discuss the day’s results, compare the results of the different team members, check the consistency of the data collection process, and identify areas for improvement for the next day. In addition to audio-recording, research team members recorded field notes about the discussion content, tone, and context of FGD activities.

### Participant recruitment and data collection methods

#### Round one

FGDs were conducted in 4 districts across Rwanda: Nyarugenge (Kigali city), Bugesera (Eastern Province), Rubavu (Western Province), and Burera (Northern Province). The fifth project district, Nyamasheke (Western Province), was not included as saturation was reached before field work began there.

Round one sought to capture the context of experiences with ANC service delivery, perceptions of benefits and limitations with current ANC services, and perceptions of the feasibility and acceptability of group care. We purposively recruited women of reproductive age who had a child under 12 months of age and had attended at least one ANC visit during the previous pregnancy. We conducted 4 FGDs among 40 women between 18 and 21 years of age, identified as “young women” or “YW” in the results section. We also conducted 4 FGDs (one in each of 4 districts) among 48 women over 21 years of age, identified as “women” or “W” in the results section. Women were purposively invited to participate to have a variety of demographic characteristics with the aim of garnering diverse perspectives. CHWs were asked to identify women who met the inclusion criteria in their respective villages and received instructions from health center staff, to accompany women to the location of the FGDs.

Before starting data collection, 2 qualitative researchers conducted a 3-day training for 4 moderators and 4 notetakers on FGD facilitation guides. Each moderator-notetaker pair facilitated an FGD of 10–12 participants. FGDs covered existing individual care models and the potential group model. Regarding group antenatal and postnatal care, facilitators described the potential model of group care and asked for respondents’ reflections and perceptions on the idea.

#### Round 2

All 18 study sites implementing group ANC and PNC were purposively sampled to include 3 health centers with the highest group visit attendance rates and 3 with the lowest attendance rates: one in Burera district, 2 in Bugesera district, and 3 in Nyamasheke district. These 6 focus groups included 56 women who had been invited to participate in group care within the last 9 months and whose estimated due dates were at least 8 weeks in the past. A team of 2 experienced qualitative data researchers (AM and BNR) facilitated FGDs across all sites.

Round 2 qualitative work sought to capture reasons women chose to attend or not attend group ANC and PNC visits, as well as soliciting suggestions to strengthen the program. Once sites were identified, the study team generated a list of women whose expected delivery date had passed and how many group visits she had attended. Field Coordinators invited women to attend FGDs by phone or by asking CHWs to contact them in person. Attempts were made to organize women into groups of high or low attenders, although this effort was not successful as most individuals had incomplete study records at that time.

### Analysis and interpretation

After completion of data collection, audio files were transcribed verbatim and cleaned by members of the research team. During round one data analysis, all FGD notes (including all verbatim quotes) were organized into thematic areas with Atlas. Ti software using a content analysis approach. Transcripts and field notes were aggregated from individual to district level and grouped to highlight themes, concepts, convergence, and diverse responses. Aggregate data were reviewed by several members of our research team (AM, BNR, DN, SM, TL) to identify key findings related to the study objectives. Representative, verbatim quotes were selected to illustrate key findings and translated from Kinyarwanda to English for results dissemination. In Round 2, transcripts were translated from Kinyarwanda to English by a professional translator. Data were organized manually into thematic areas following content analysis approach by TL and reviewed by AM and SM.

### Ethical consideration

Ethical approval for both rounds was granted by the Rwanda National Ethics Committee and University of California, San Francisco Institutional Review Board. A written informed consent was obtained from each participant prior to starting the FGD. Participants were given the opportunity to read the consent form in Kinyarwanda and illiterate participants had it read to them by a witness. Only participants who agreed to participate and signed the consent form were invited to join the FGDs. Moderators obtained permission from study participants to record the conversation and voluntary participation was ensured throughout the study. All information was kept confidential and personal identifiers were not recorded on audio and written files.

## Results

Resulting themes are organized into those that emerged from the pre-implementation phase of data collection, and those that emerged from data collection at the mid-point of the group ANC and PNC trial. A summary of these themes and sub-themes appears in Table 1.

**Table 1:** Themes and sub-themes in focus group discussions among reproductive-aged women both before and after implementation of group antenatal care (ANC) and postnatal care (PNC) in Rwanda

Timepoint	Themes	Sub-themes	Details
Before implementation	Group ANC may offer advantages for pregnant women	Social cohesion	Friendship; Shared accountability for perinatal outcomes in the community
		Shared knowledge	Overcome the fear of asking questions or disclosing concerns
	Some women may be too afraid to share personal information in a group		Test results and measurements should be confidential
After implementation	Group ANC is better than individual ANC	Problem-solving and increased health literacy as a group	Increased participant knowledge about ANC interventions, nutrition, danger signs, planning for facility birth
		New and meaningful relationships form	Among pregnant women and between providers and pregnant women
	The group model of care cannot overcome all barriers to ANC and PNC attendance	Financial barriers are significant	Co-payments required at the time of care; lost wages; important work at home unattended while woman attends ANC
		Distance between home and facility	Primary means of transportation is walking; other transportation is expensive
		Women forget appointment dates/times	An improved appointment reminder method is needed
		Shortage of staff at health centers results in long wait times in both models of care	Some group visits were abandoned by the provider who was called out to attend to a woman in labor
		Women, family members, and communities do not value PNC	Women expect a reward for “completing” the program

### Before implementation of group ANC and PNC

#### Group ANC may offer advantages for pregnant women

Before group ANC and PNC were implemented, women anticipated multiple potential benefits of participation of this alternative model of care, including increased social cohesion, learning from the experiences of their peers, and ease of expression. The verbatim quote below reveals that women do not always get answers to their health-related questions in the individual model of care, and that they are better able to learn and solve health-related problems by participating in group care.

*You can have a problem and then you try to resolve it but you don’t get the solution. But as we will be in group, you can share that problem with your group members who may advise you. Especially when you come for ANC, you have so many questions to ask; and when you want to ask to the healthcare provider, they don’t have enough time to answer.* (Burera district, YW P7)

FGD participants hypothesized that group sharing could help women “open up” about questions and concerns with a resulting increase in health education.

*The advantage of the groups is that sometimes women who go for ANC for the first time are afraid to tell everything to the healthcare provider due to ignorance but when she is with other women in groups, she tells them her problems and they advise her.* (Burera district, W P12)

#### Fear of sharing personal information

Enthusiasm for the group care model was tempered by the impulse to protect one’s privacy.

*All people don’t have the same understanding. When you are alone with the healthcare provider, you tell them many things so that they may help you but when you are with many people, you hide some of the things relative to your disease and how you feel.* (Bugesera district, W P6)

Interestingly, one woman was concerned by the idea that during group health assessments her peers could notice her weight measurement and gossip about it:

*The woman can tell everyone your weight and comment on you [all the focus group participants laugh].* (Bugesera district, YW P1)

Another woman elucidated both the fear and the hope associated with sharing one’s personal information in a group of peers:

*Sometimes you may be HIV infected and when you share your secret you will start thinking that everyone will know about it. Yet it is also good when you dare share it, it releases you from that load, and if they are informed, they may know how to behave towards you."* (Burera district, W P10)

### After group care implementation

We did not find any significant differences in the qualitative data content between FGDs at sites that reported the highest rates of attendance and those that reported the lowest rates of attendance. The main themes that emerged from these FGDs were that 1) group ANC is better than individual ANC, and 2) some barriers to ANC attendance are not addressed by the transition to the group model of ANC delivery.

#### Group ANC is better than individual ANC

Many women described a significant increase in health-related and self-care knowledge related to group care participation.

*With the old way of consultation, the nurse would only do her job, and when she is done, you would go home. But in the group, there was something extraordinary—that of sitting and asking the nurse what she was doing, and she would take time to explain to you. You would also ask more experienced mothers in your group whether they may have gone through such or such other experience. They would also relate to you what they saw and how they solved any complicated situation.* (Nyamasheke district, HC1, P6)

Women reported forming meaningful relationships with their pregnant peers and their providers:

*The consultation in group care improves the relationships among people. For example, I didn’t know this woman before. Today I cannot pass by her without greeting her; she may even help me when I’ve got a problem and fail to remember what I can do about it; in that case I can feel free to ask her."* (Nyamasheke district, HC1, P3)

*Some mothers who were not yet in the group care . . . were surprised at seeing the nurse come and sit near me, and then ask me about my health and my child’s health. They eagerly inquired why she was much interested in me only to learn that we got to know each other when she was training us in the group care. Therefore, I found that there is a difference, and this led me to like the program much more and attend it.* (Burera district, HC1, P7)

#### The group model of care cannot overcome all barriers to ANC and/or PNC attendance

None of the women who participated in the focus groups convened after implementation mentioned that concerns about privacy impeded their group care participation. The barriers to group care participation most commonly cited were lack of the financial resources required to attend, family or community members who actively discourage attendance, and distances that must be travelled on foot. Some women couldn’t afford to lose wages:

*[One may decide to stay] at home because a person may use that time to go and make a thousand Rwandan Francs to sustain her family.* (Burera district, HC1, P6)

Some women can’t afford the payment required at the time of service:

*It sometimes happens to a person to lack that amount of money of 200 Rwandan Francs we pay for the form which is filled for a woman who has come for consultation. This may also be a reason for some people to fail to attend! . . . When you don’t have this money, you can go to a friend and borrow. You may even stop buying salt provided that you bring [the money]. It is an obstacle [to ANC attendance].* (Nyamasheke district, HC1, P4)

Multiple women at one health center in Bugesera district reported that their husbands or others in the community discouraged them from attending ANC and PNC.

*Your husband may feel annoyed by the number of times you go to the health center; and when he has compared them to what he may see other women do—like weeding their crops—he may order you not to go there once again.* (Bugesera district, HC1, P3)

Women also commonly reported that the long distance many must walk to the health center is a barrier to timely group care attendance.

*All of us [in the same group] didn’t arrive here at the same time because of different distances we have to walk. A long distance can also discourage a person from coming here for consultation or tests.* (Nyamasheke district, HC3, P2)

When asked for suggestions to improve women’s experience of group ANC and group PNC, women did not make any recommendations related to the fundamental components of the model, such as health assessments shared in the group space or facilitated group discussion activities or topics. Some of their suggestions were related to solving problems that might impede attendance, and others noted that the logistics of starting and ending a group visit “on time” were complicated. A common response was that a better system is needed to remind women of the appointments, especially among those women who cannot read.

Another common message was that more community outreach is needed to help male partners, female next-of-kin, older women in the community, and pregnant and postnatal mothers understand the purpose and value of ANC and PNC in general, and group care in particular.

*I can suggest that this topic [group ANC and PNC] should be made part of discussions we have during the parents’ evening [regular community meetings] where we may be together with our husbands. There, they may hear about it; or, as it was said, it can be made an obligation and be published to people as they publish other public meetings of local government.* (Bugesera district, HC1, P6)

Finally, participants were well aware of the challenges of introducing scheduled appointments at a specific date and hour in a system that has not previously used this method to allocate both provider and client time. Several women pointed out that all participants must be acculturated to a new way of organizing their time and that more staff are needed so that group care facilitators would not have to cross-cover multiple services simultaneously (for example, ANC, maternity, and family planning).

*There is something which needs correction for both of us beneficiaries and trainers. We did not keep time when we would come to the health center for group care. On our side, some of us would come on time and be bound to wait for the latecomers . . . the nurse also would fail to attend the group because of other clients she had to help. So, I would suggest that they may increase the number of nurses to help the other mothers so that the [provider] may be available on time. Group care members also should learn to keep time.* (Burera district, HC1, P9)

The postnatal component of the group care model in Rwanda was conceived as a way to introduce the 6-week postnatal visit to providers and mothers in a system that did not yet have a mature postnatal care package. When women were asked about their perceptions of group postnatal care, they reported a mix of confusion about the purpose of the visit, disappointment that they didn’t receive a gift for having completed the series of visits, and contentment when providers reassured them that their children were growing well.

## Discussion

Despite the fact that women voiced some concerns about the group care model before implementation, women who actually participated in group ANC and PNC in Rwanda report positive experiences of increased information-sharing and learning augmented by the friendship and support of peers and providers. In fact, one barrier to ANC mentioned in pre-implementation FGDs was that providers treat women poorly; whereas all reports of provider behavior in the post-implementation FGDs suggested mutual respect and warmth. Our results are consistent with other published reports of group care. A recent systematic review offers a synthesis of how group ANC programs in LMICs have been designed and implemented [22], and several of those programs include evaluations and study protocols that have been published [16, 18, 23–29]. Table 2, included as Additional file 1, summarizes published reports of outcomes when standard, individual-visit ANC has been compared to group ANC in LMICs.

Only 2 other qualitative reports of perceptions of the feasibility and acceptability of group ANC in low- and middle-income countries are available. In a recently published feasibility study focused on group ANC in 3 facilities in an Indian urban center, health care providers and health care consumers were interviewed after watching a single demonstration of a group ANC visit [30]. Providers and women responded positively to all aspects of the group ANC demonstration they watched, while the providers stated that support at the facility level would be necessary for successful implementation and women stated that pre-visit reminders by text or phone call might encourage attendance. These results are consistent with suggestions from participants in our study, who recommended that a system for phone reminders be implemented and that provider time be allocated so that groups could start and end on time. Qualitative results were included in the report of a group ANC and PNC pilot in both Tanzania and Malawi [31]. In that study, providers and participating women liked the egalitarian relationships they developed and the amount and quality of health education shared in a facilitated group discussion. These themes also emerged in our study.

Some qualitative results obtained 9 months after implementation were unexpected. We were interested to note that none of the focus group discussion participants noted any concerns about privacy, which had been a consistent theme before implementation. This could be because group care facilitators took care to establish expectations of confidentiality and trust before every group discussion, as they were trained to do. However, it is possible that women with specific concerns about privacy and confidentiality in group care may have declined to participate in focus groups. We plan to follow up further with women who refused to participate in the trial at all to learn more about their decision-making process. We also learned that the expectation of a material reward for attending the postnatal visit and “completing the program” was quite widespread. Because this trial did not offer material rewards to providers or participants, there could be some disappointment that we will work to understand and mitigate.

We learned that there is still a gap between the value the health system believes ANC and PNC (including both individual and group ANC and PNC) offer families and the perceived value of these services among beneficiaries. Multiple comments from focus group participants suggest that for some families, the costs (such as lost wages) of engaging in preventative services are too high to justify participation. Whether or not group ANC and PNC can augment the perceived value enough to overcome these cost concerns is an unanswered question, but it is a question that we expect this trial’s quantitative results will help answer. Members of our team who are local experts in community outreach, including public health messaging from the Ministry of Health, continue to work toward meeting the informational needs of the community with respect to the purpose and value of ANC and PNC.

Suggestions for program improvement made by focus group participants included decreasing the time women spent waiting for each other and for providers to arrive at the group care session, creating communications systems for reminding families about appointment dates and times, and increasing community outreach. Increased community outreach, while complex and somewhat costly, fits within the plans and priorities of the public health system and is most feasible to implement. This community outreach strategy should be informed by future data collection among male partners. Providing more effective appointment reminders are more challenging to implement, as the existent systems do not support these. Ensuring that providers can conduct group visits in a timely manner, without simultaneous demands for their time from other services at the health center, requires an ongoing discussion with administrators about staff allocation patterns and staff shortages. Further discussions with administrators at every level of the health system will be required to incorporate this feedback into any future plans to spread and scale the group care model in Rwanda.

### Limitations

Because the baseline qualitative work did not include women in Nyamasheke district, it is possible that we missed themes or sub-themes unique to their pre-implementation ideas about the group care model. However, women in Nyamasheke district were represented in 3 FGDs after implementation.

## Conclusion

Group ANC and PNC provide compelling benefits to women and families, although the proportion of families who are better-served by this innovative model of care is unknown. If the model requires the addition of human resources at the health center, intensive reminder communications, and large-scale community outreach to benefit the largest number of pregnant and postnatal mothers, those additional resources required must be factored into any future decision to transition to group care in Rwanda. We will use these findings to follow up with another set of qualitative data collection activities at the end of the trial.

## Additional file

Additional file 1: Table 2 Published studies of group antenatal care in Low- and Middle-Income Countries that report quantitative results. (DOCX 24 kb)

## Data Availability

Data sets will be made accessible and open after the end of the parent study and publication of the primary results, according to the Bill and Melinda Gates Foundation Open Access policy. The study instruments used during this qualitative study are available from the corresponding author on reasonable request.

## Abbreviations

ANC: Antenatal care
CHW: Community health worker
FANC: Focused antenatal care
FGD: Focus group discussion
LMIC: Low- and middle-income country
PNC: Postnatal care
USD: United States dollars
