# Supplementary material for: Before and after implementation of group antenatal care in Rwanda: a qualitative study of women’s experiences
Source: Reprod Health. 2019 Jun 27;16:90. doi: 10.1186/s12978-019-0750-5 (PMC6595554; doi:10.1186/s12978-019-0750-5)
Supplement: Supplementary file 1 — Table 2 Published studies of group antenatal care in Low- and Middle-Income Countries that report quantitative results. (DOCX 24 kb) [file 12978_2019_750_MOESM1_ESM.docx]

Additional file 1

Table 2. Published studies of group antenatal care in Low- and Middle-Income Countries that report quantitative results

| Reference | Location | Study design | Population | Sample size (number of participants included in study) | Selected outcomes of interest | Result | p value |
| --- | --- | --- | --- | --- | --- | --- | --- |
| Arnold J et al.^19^ | Botswana | Descriptive; no comparison group | Men who had attended at least 5 group antenatal care sessions  with their pregnant partners at one private hospital | Convenience sample of 7 men | Perceptions of group antenatal care | Mean score 113.0 ± 8.29 out of a possible 125 | Not applicable |
|  |  |  |  |  | Satisfaction with group antenatal care | Mean score of 13.43 ± 1.72 out of a possible 15 | Not applicable |
| Jafari F et al, 2010^23^ | Iran | Cluster randomized controlled trial (14 health centers, intent-to-treat) | Pregnant women, average age 26 years old, 47% nulliparous 48% had completed high school or a higher education level | Women in group antenatal care, n=344;  women in individual antenatal care, n=334 | Preterm birth | 6.3% preterm birth rate among women at group antenatal care sites versus 9.7% preterm birth rate among women at individual antenatal care sites | .191 |
|  |  |  |  |  | Low birth weight | 6.3% low birth weight rate among women at group antenatal care sites versus 9.1% low birth weight rate among women at individual antenatal care sites | .213 |
|  |  |  |  |  | Intrauterine growth restriction | 2.2% intrauterine growth restriction rate among women at group antenatal care sites versus 3% intrauterine growth restriction rate among women at individual antenatal care sites | .541 |
|  |  |  |  |  | Mean birth weight (g) | 3248.1 g among women at group antenatal care sites versus 3160.3 g among women at individual antenatal care sites | .011 |
|  |  |  |  |  | Perinatal death | 2.2% perinatal death rate among women at group antenatal care sites versus 3.2% perinatal death rate among women at individual antenatal care sites | .432 |
| Jafari et al, 2010^16^ |  |  |  | Women in group antenatal care, n=344;  women in individual antenatal care, n=334 | Satisfaction with care | Difference in mean item score: women in group care were very satisfied with prenatal care services versus women in individual care group were somewhat satisfied | < 0.000 |
| Lori J et al.^21^ | Ghana | Individual randomized controlled trial in one urban hospital | Pregnant women: average age approximately 28 years old, 32.9% completed any high school or higher education | Women in group antenatal care, n=120; women in individual antenatal care, n=120 | Health literacy | Overall higher number of self-care measures and danger signs recalled by women in group care versus women in individual care | 0.01 |
|  |  |  |  |  |  | Women in group care more likely to report a making plan for emergency transport and saving money for birth versus women in individual care | 0.001 |
|  |  |  |  |  |  | Women in group care demonstrated greater BNReastfeeding knowledge versus women in individual care | <0.01 |
|  |  |  |  |  |  | Women in group care were more likely to report they planned to use a family planning method | 0. 01 |
| Patil CL et al.^24^ | Malawi and Tanzania | Individual randomized controlled trial in 4 sites: 2 in Malawi (rural) and 2 in Tanzania (urban) | Pregnant women: in Tanzania, about half the women were Muslim, while only one woman in Malawi was Muslim; women in Tanzania had more education, were less likely to be farmers, and had more food security and family assets. | Women in group antenatal care in Malawi, n=54; Women in group individual care in Malawi, n=58. | Pregnancy-related empowerment | Malawi: mean score 59.1 among women at group antenatal care sites versus 43.7 among women at individual antenatal care sites   \|  \|  \|  \| \| --- \| --- \| --- \| | < 0.0001 |
|  |  |  |  | Women in group antenatal care in Tanzania, n=56; Women in group individual care in Tanzania, n=50. |  | Tanzania: mean score 51.4 among women at group antenatal care sites versus 50 among women at individual antenatal care sites  difference | 0.305 |
| Patil CL et al.^18^ |  |  |  | Women in group antenatal care in both countries, n=110; women in individual care in both countries, n=108 | Attended at least 4 ANC visits | 94.1% of women randomized to group antenatal care versus 58.2% of women randomized to individual antenatal care | *<*0.001 |
|  |  |  |  |  | Attended a postnatal care visit (6 weeks after birth) | 74.5% of women randomized to group antenatal care versus 50.0% of women randomized to individual antenatal care | *<*0.001 |
|  |  |  |  |  | Satisfaction with care | 39.2 mean score among in group antenatal care versus 27.7 mean score among women in individual antenatal care | *<*0.001 |
| Ruhl L.^17^ | Kenya, Teso North District | Retrospective evaluation comparing outcomes at 5 facilities that implemented a group antenatal care model and 10 comparison health facilities (standard, individual antenatal care) | Not reported | Not reported | Average monthly number of new family planning (FP) visits | 41.5 visits at group care facilities compared to 32.3 visits at individual care facilities | 0.004 |
|  |  |  |  |  | Median monthly number of “longterm” FP visits | 18 visits at group care facilities compared to 11 visits at individual care facilities | 0.001 |
|  |  |  |  |  | Median monthly number of newborns with low birth weight | Zero newborns with low birth weight at group care facilities compared to one newborn with low birth weight at individual care facilities | 0.001 |
|  |  |  |  |  | Mean monthly number of the uptake of 4 or more ANC visits | No significant difference |  |
|  |  |  |  |  | Mean monthly number of facility deliveries | No significant difference |  |
|  |  |  |  |  | Oral polio vaccine doses | No significant difference |  |
